# Cessation of smoking and drinking and the risk of laryngeal cancer

**DOI:** 10.1038/sj.bjc.6600638

**Published:** 2002-11-12

**Authors:** A Altieri, C Bosetti, R Talamini, S Gallus, S Franceschi, F Levi, L Dal Maso, E Negri, C La Vecchia

**Affiliations:** Istituto di Ricerche Farmacologiche ‘Mario Negri’, Milan, Italy; Servizio di Epidemiologia, Centro di Riferimento Oncologico, Aviano, Italy; International Agency for Research on Cancer, Lyon, France; Registre Vaudois des Tumeurs, Institut Universitaire de Médicine Sociale et Préventive, Lausanne, Switzerland; Istituto di Statistica Medica e Biometria, Università degli Studi di Milano, Milan, Italy

**Keywords:** laryngeal cancer, alcohol drinking, smoking cessation, risk factors, case–control study

## Abstract

A case–control study was conducted in Italy and Switzerland between 1992 and 2000 on 527 cases of laryngeal cancer and 1297 hospital controls. The risk of laryngeal cancer steadily decreased from 3 years after stopping smoking. Some decline in risk was observed only 20 years or more after stopping drinking.

*British Journal of Cancer* (2002) **87**, 1227–1229. doi:10.1038/sj.bjc.6600638 www.bjcancer.com

© 2002 Cancer Research UK

## 

Cancer of the larynx is extremely rare in individuals who neither smoke nor drink alcohol (Austin and Reynolds, 1996). The risk rises with increasing levels of smoking and drinking, each agent approximately multiplying the effects of the other (Elwood *et al*, 1984; Blot, 1992; Dosemeci *et al*, 1997; Talamini *et al*, 2002). The risk of laryngeal cancer is 10–20-fold higher in current smokers than in non-smokers (Tuyns *et al*, 1988; Talamini *et al*, 2002), and it appears to decline steeply with time since stopping smoking (Wynder and Stellman, 1977; Tuyns *et al*, 1988; Falk *et al*, 1989; Franceschi *et al*, 1990; Zatonski *et al*, 1991). Thus, in a multicentric case–control study in four European countries (Tuyns *et al*, 1988) a 60% decreased risk was observed in subjects who had stopped smoking for 5–9 years, and a 70% decrease in those who had stopped for 10 or more years. In addition, in a case–control study from Italy, the odds ratio (OR) for ex-smokers after 10 or more years was about one-third of that of current smokers (Franceschi *et al*, 1990).

An independent role of alcohol has been reported by several studies (Iarc, (1988); Falk *et al*, 1989; Doll *et al*, 1999; Bagnardi *et al*, 2001), including a few restricted to non-smokers (Burch *et al*, 1981; Elwood *et al*, 1984; Tuyns *et al*, 1988; Bosetti *et al*, 2002). The risk appears to rise steeply with increasing level of alcohol intake, in the absence, however, of a clear pattern with duration of the habit or age at starting (Bosetti *et al*, 2002; Talamini *et al*, 2002). Little is known on the time–risk relation of stopping drinking on laryngeal cancer.

To elucidate the separate and combined effect on laryngeal cancer risk of time after smoking and drinking cessation, we analysed data from a multicentric case–control study conducted in Italy and Switzerland (Talamini *et al*, 2002). In this study a 20-fold higher risk was found for current smokers, and the risk was approximately 40-fold higher for heavy smokers (i.e. ⩾25 cigarettes/day). With reference to alcohol consumption, the risk increased in relation to number of drinks, with a six-fold increase for ⩾8 drinks/day.

## PATIENTS AND METHODS

A case–control study of cancer of the larynx was conducted between 1992 and 2000 in two areas of northern Italy (the provinces of Pordenone and the greater Milan area), and in the Swiss Canton of Vaud (Talamini *et al*, 2002). Cases were 527 patients (478 men and 49 women, median age 61 years, range 30–79) admitted to the major teaching and general hospitals in the areas under study with histologically confirmed squamous-cell carcinoma of the larynx, diagnosed no longer than 1 year before the interview. Laryngeal cancer cases included 271 glottis, 117 supraglottis, and 139 other or unspecified laryngeal cancers. Controls were 1297 subjects (1052 men and 245 women, median age 61 years, range 30–79) frequency-matched with cases by 5-year age groups, sex and study centre, selected among patients admitted to the same hospitals as cases for a wide spectrum of acute, non-neoplastic conditions, not related to smoking, alcohol consumption or long-term modifications of diet. To compensate for the rarity of laryngeal cancer in women, a control-to-case ratio of about five was chosen for females, as opposed to two for males. Twenty-seven per cent of the controls were admitted for traumas, 22% for other orthopaedic disorders, 29% for acute surgical conditions and 23% for miscellaneous other illnesses, including eye, ear, nose and throat, skin or dental disorders.

Cases and controls were interviewed by *ad hoc* trained interviewers during their hospital stay using a structured questionnaire. Information was collected on socio-demographic characteristics, anthropometric measures, lifestyle habits, including tobacco smoking and alcohol drinking, dietary habits, personal and family medical history. The section on smoking included questions on smoking status (never, current, or ex-smokers), daily number of cigarettes/cigars smoked in different life periods, age at starting, duration of the habit and, for ex-smokers, age at smoking cessation. Information on alcohol consumption included the average number of days per week for each type of alcoholic beverage consumed (wine, beer and spirits), the average number of drinks per day and, for ex-drinkers, age at drinking cessation. Ex-drinkers and ex-smokers were subjects who had abstained from any type of drinking or smoking for at least 12 months.

Odds ratios (OR) and the corresponding 95% confidence intervals (CI) were estimated using unconditional multiple logistic regression models (Breslow and Day, 1980), including terms for age, sex, study centre, years of education, and, when appropriate, alcohol and tobacco consumption.

## RESULTS

Table 1

**Table 1:**
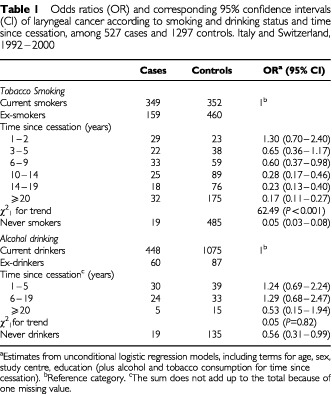
Odds ratios (OR) and corresponding 95% confidence intervals (CI) of laryngeal cancer according to smoking and drinking status and time since cessation, among 527 cases and 1297 controls. Italy and Switzerland, 1992–2000

gives the distribution of the 527 cases of laryngeal cancer and the 1297 control subjects, according to smoking and drinking status, and time since cessation. A total of 159 cases were ex-smokers and 60 were ex-drinkers. Compared to current smokers, the OR was slightly above unity in the 2 years after smoking cessation, declined to 0.60 up to 9 years, to around 0.25 for 10 to 19 years, and to 0.17 for 20 years or more after cessation. Despite the decline, the risk 20 years after stopping smoking was still greater than that for never smokers (OR=0.05). The inverse trend in risk with time since smoking cessation was significant (*P*<0.001).

In relation to time since stopping alcohol drinking, no consistent pattern of risk was observed up to 20 years, and some risk reduction was evident only 20 years since stopping the habit. The ORs for ex-drinkers, as compared to current drinkers, were 1.24 for 1–5 years since drinking cessation, 1.29 for 6–19 years. The OR was 0.53 among the few subjects who had stopped drinking for 20 or more years, i.e., similar to that of never drinkers (OR=0.56). None of these estimates, nor the trend in risk, was significant.

Table 2

**Table 2:**
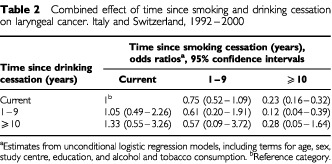
Combined effect of time since smoking and drinking cessation on laryngeal cancer. Italy and Switzerland, 1992–2000

shows the combined effect of stopping tobacco smoking and alcohol drinking, in relation to time since cessation of each habit. Compared to subjects who currently smoked and drank, the risk of laryngeal cancer decreased with time since smoking cessation across all drinking strata. Conversely, no decline in risk was found for drinking cessation among current smokers or ex-smokers. The OR for laryngeal cancer among those who had quit both habits for 10 or more years was similar to that of subjects who had quit smoking but remained current drinkers (OR=0.28).

## DISCUSSION

Our study, based on a large dataset on laryngeal cancer, is one of the few to examine the effect on risk of stopping both smoking and drinking. It confirms that the favourable impact of stopping smoking is already evident in the few years after cessation, and that laryngeal cancer risk is reduced by over 70% ten or more years after stopping smoking. Such a risk reduction has been observed also for oesophageal cancer (Castellsagué *et al*, 1999; Bosetti *et al*, 2000), and is similar, or if anything stronger, to that recognised for lung cancer (Peto *et al*, 2000; Stellman *et al*, 2001).

Some favourable impact of stopping drinking may become apparent in the long term, but it is difficult to estimate on account of the small number of subjects who had stopped drinking for 20 years or more. The persistence of an excess risk up to several years after stopping drinking indicates that alcohol is probably not only a late stage carcinogen (Day and Brown, 1980), as previously observed for oral, pharyngeal (Franceschi *et al*, 2000), and oesophageal (Bosetti *et al*, 2000) cancers. Furthermore, the persistence of exposure to tobacco among ex-drinkers may play an important role in limiting the benefits of drinking cessation.

This pattern of risk after drinking cessation may be also due to certain characteristics of the selected group of people who had stopped drinking (8% of control subjects). Most former drinkers had, in fact, higher alcohol consumption than current drinkers (median number of drinks per week was 42 in ex- and 28 in current drinkers), and it is possible that health related conditions had affected the decision to stop alcohol consumption, though this aspect was not investigated. In any case, it is unlikely that early symptoms of cancer of the larynx had accounted for drinking cessation, since the excess risk persisted up to 20 years prior to cancer diagnosis.

In this study information on smoking and alcohol was satisfactorily reproducible and valid (Ferraroni *et al*, 1996), although no information was available on the reliability of data on time-related aspects of the two habits. The hospital setting should, however, ensure a reasonable comparability of information collected (D'Avanzo *et al*, 1996), and careful allowance was made for tobacco and alcohol, as well as education.

In the present study, 90% of laryngeal cancer was attributable to tobacco, while alcohol explained 58% of cases. Together, these two factors were responsible for 96% of laryngeal cancers (Bruzzi *et al*, 1985; Talamini *et al*, 2002). Stopping tobacco smoking, and possibly alcohol drinking, could therefore have a substantial impact in reducing laryngeal cancer risk. At public health level, however, stopping smoking remains the key measure to control laryngeal cancer.
